# Automated detection and segmentation of intracranial hemorrhage suspect hyperdensities in non-contrast-enhanced CT scans of acute stroke patients

**DOI:** 10.1007/s00330-021-08352-4

**Published:** 2021-11-13

**Authors:** N. Schmitt, Y. Mokli, C. S. Weyland, S. Gerry, C. Herweh, P. A. Ringleb, S. Nagel

**Affiliations:** 1 Department of Neuroradiology, Heidelberg University Hospital, Heidelberg, Germany; 2 Department of Neurology, Heidelberg University Hospital, Heidelberg, Germany; 3 Department of Psychiatry and Psychotherapy, Giessen and Marburg University Hospital, Giessen, Germany; 4 Centre for Statistics in Medicine, University of Oxford, Oxford, UK

**Keywords:** CT, Acute stroke, Hyperdense volume, Blood, Artificial intelligence

## Abstract

**Objectives:**

Artif icial intelligence (AI)–based image analysis is increasingly applied in the acute stroke field. Its implementation for the detection and quantification of hemorrhage suspect hyperdensities in non-contrast-enhanced head CT (NCCT) scans may facilitate clinical decision-making and accelerate stroke management.

**Methods:**

NCCTs of 160 patients with suspected acute stroke were analyzed regarding the presence or absence of acute intracranial hemorrhages (ICH) using a novel AI-based algorithm. Read was performed by two blinded neuroradiology residents (R1 and R2). Ground truth was established by an expert neuroradiologist. Specificity, sensitivity, and area under the curve were calculated for ICH and intraparenchymal hemorrhage (IPH) detection. IPH-volumes were segmented and quantified automatically by the algorithm and semi-automatically. Intraclass correlation coefficient (ICC) and Dice coefficient (DC) were calculated.

**Results:**

In total, 79 of 160 patients showed acute ICH, while 47 had IPH. Sensitivity and specificity for ICH detection were 0.91 and 0.89 for the algorithm; 0.99 and 0.98 for R1; and 1.00 and 0.98 for R2. Sensitivity and specificity for IPH detection were 0.98 and 0.89 for the algorithm; 0.83 and 0.99 for R1; and 0.91 and 0.99 for R2. Interreader reliability for ICH and IPH detection showed strong agreements for the algorithm (0.80 and 0.84), R1 (0.96 and 0.84), and R2 (0.98 and 0.92), respectively. ICC indicated an excellent (0.98) agreement between the algorithm and the reference standard of the IPH-volumes. The mean DC was 0.82.

**Conclusion:**

The AI-based algorithm reliably assessed the presence or absence of acute ICHs in this dataset and quantified IPH volumes precisely.

**Key Points:**

• *Artificial intelligence (AI) is able to detect hyperdense volumes on brain CTs reliably.*

• *Sensitivity and specificity are highest for the detection of intraparenchymal hemorrhages.*

• *Interreader reliability for hemorrhage detection shows strong agreement for AI and human readers.*

**Supplementary Information:**

The online version contains supplementary material available at 10.1007/s00330-021-08352-4.

## Introduction

Acute stroke accounts for almost 10% of all deaths worldwide. It affects one in four people over their lifetime [1], and intracranial hemorrhage (ICH) is one of the most devastating forms [2]. According to its wide availability and its low acquisition time, non-contrast-enhanced computed tomography (NCCT) of the head is the preferred imaging modality for patients with acute stroke symptoms [3]. In this context, NCCT serves—in particular—to rule out acute ICH and thus enables a faster and more adequate treatment [4].

In the last few decades, artificial intelligence (AI)–aided analysis of medical imaging data has been implemented reliably in many healthcare areas [5], including the diagnosis of acute ischemic or hemorrhagic stroke. Since these so-called computer-aided diagnosis (CAD) applications have been primarily introduced to provide radiologists with a second opinion, they have nowadays become an integral part of clinical routine [6]. One established CE-marked decision support tool on NCCT of patients with suspected acute stroke is e-ASPECTS by the company Brainomix Limited (hereinafter referred to as Brainomix). This application can assess signs of ischemic hypodensity in NCCT of the brain and quantify the associated Alberta Stroke Program Early Computed Tomography Score (ASPECTS) in nearly real time [7]. Since acute ICH accounts for approximately 10–20% of all strokes [8], an AI-based image analysis regarding the detection and quantification of hemorrhage suspect hyperdensities in patients with acute stroke would help to improve workflow, for example by triaging radiological data and thus improving outcomes [9], as well as reduce medical errors, for example by providing a second opinion [10]. Therefore, the present study investigates the performance of a novel algorithm from Brainomix regarding the automated detection and segmentation of hemorrhage suspect hyperdensities on NCCT in comparison to neuroradiologists.

## Methods

### Data collection and study design

The datasets were retrospectively assembled over a period of 1 year, starting from September 2017. All cases of patients with a suspected acute stroke presented in our department who underwent an NCCT were assessed. A total of 1297 cases were evaluated (fellow with 5 years of training in neurology and stroke) for their eligibility for inclusion within our trial testing data subset (TDS, *n* = 160). Data collection and analysis were approved by the local ethics committee (Medical Faculty of Heidelberg University). Patient consent was waived due to the retrospective, descriptive character of this single-center study.

The primary screening phase involved a visual inspection of all NCCT layers. Individual labeling was made with annotation about the following: (1) the presence or absence of ICH, and if present its type and anatomical localization; (2) the presence of pronounced artifacts, their localization, and probable cause (e.g., coils, clipping, or hearing aids); (3) the presence of pronounced physiological or pathological calcifications with a descriptive quantification; and finally (4) the presence of other hemorrhage-like structures (for example, meningioma, hyperdense vessels, calcified tumors, and vascular malformations).

All the included cases were pseudonymized and ranked according to the date and time of admission to our hospital. During this process, 110 cases were excluded; principal exclusion criteria were age under 18 years old, cases with absent, deficient, or inaccurate clinical or radiological findings, as well as reduced quality of NCCT images with a resulted uncertain interpretability usually arising due to different types of pronounced artifacts, like artifacts related to excessive motion or dense foreign bodies such as endovascular coils and hearing aids [11].

In the subsequent sampling phase, based on the pre-calculated sample size, we tried to generate a challenging evaluation dataset using a stratified-convenience sampling strategy. In order to enrich the cohort with challenging pathologies of the algorithm, we selectively formed two main groups—with serval subgroups each—based on the presence or absence of ICHs, type of hemorrhagic lesions, and the appearance of hemorrhagic-like lesions (see Table 1 in the Supplementary Appendix). The sampling was terminated whenever the targeted number of cases in each group was reached. Detailed information on the inclusion criteria as well as the sampling process is provided in Fig. 1 of the Supplementary Appendix.

Significant clinical findings were gathered retrospectively from the electronic medical charts and documents; including basic demographics (age, gender), date and time of admission and first CT imaging, representative scores (pre-stroke Rankin Score [pmRS], National Institutes of Health Stroke Scale [NIHSS], arterial pressure), use of anticoagulants or antiplatelet medications, and the presence of relevant co-morbidities (arterial hypertension, diabetes mellitus, hypercholesterinemia, previous stroke, and atrial fibrillation or atrial flutter).

### Imaging andpostprocessing

CT image acquisition of the 160 patients was performed in a non-enhanced technique with standard settings according to clinical routine. Therefore a 64-slice multidetector, single-source scanner (Somatom Definition AS, Siemens Healthineers) with a tube voltage of 120 kV and a tube current of 20 mAs was used. Reconstruction of all CT images was conducted with a J40s kernel with a slice thickness of 1 mm in the axial plane.

The hyperdensities detection and segmentation algorithm from Brainomix was developed by training a deep convolutional neural network (CNN) on thin-slice CT brain images with manual voxel-wise annotations of hyperdensities, as well as images from a normal control population. Fully convolutional networks and, in particular, U-Net were designed to make predictions at the voxel level. U-Net was initially proposed for biomedical image segmentation [12]. A simplified description of its architecture can be comprehended as an encoder network followed by a decoder network. The encoder is able to capture the context in the image into deeper features, whereas the decoder enables precise localization of those features into the voxel space. The developed U-Net operates into the 3D volumes in order to segment the hyperdense regions from the CT scans in a fully automated manner. Voxel-wise probabilities of hyperdensity are then thresholded to produce a binary hyperdense volume mask and corresponding volume estimate (in ml). No Hounsfield unit-based thresholds are being used to distinguish hyperdensities typical of acute bleed from other hyperdensities, such as calcification. Instead, the CNN is trained with cases that have common calcification (e.g., pineal gland and choroid plexus) and those voxels do not belong to the positive class and therefore are trained to be recognized as normal. CT images of our TDS were analyzed fully automatically by the algorithm regarding the presence or absence of hemorrhage suspect hyperdensities. Additionally, the intracranial located hyperdensities were automatically segmented and quantified by the algorithm. The algorithm generates all the results in nearly real-time (up to a maximum processing time of 1 min).

**Fig. 1:**
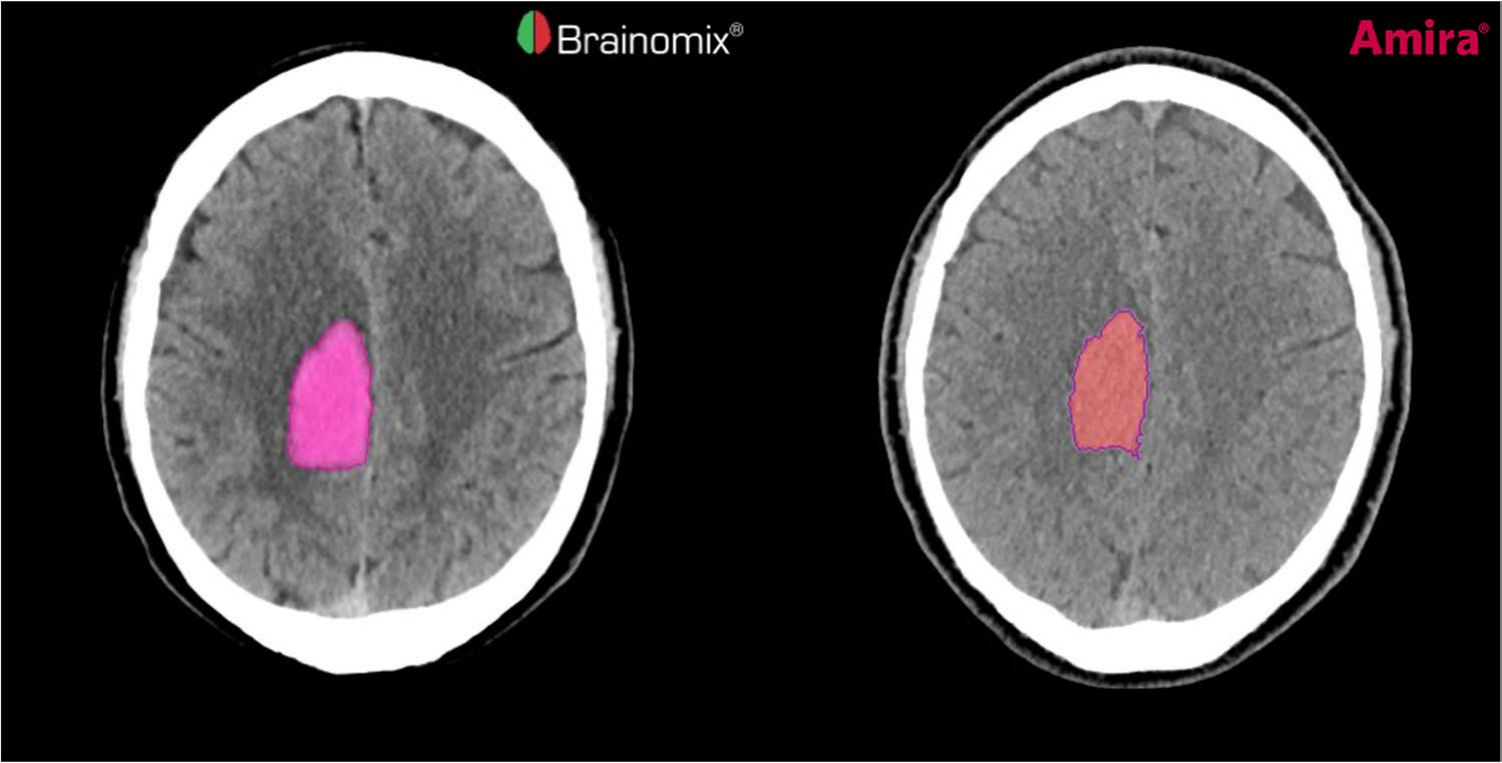
Segmentation of an intraparenchymal hemorrhage (IPH) in the right frontal and parietal lobe of a patient with acute stroke. The IPH was segmented automatically (left) by the algorithm from Brainomix and semi-automatically (right) by Amira software to provide a reference standard. The Dice similarity coefficient for this example was 0.95

All CT scans were read by two neuroradiology residents with 2 years of experience each, using a picture archiving and communication system workstation (CENTRICITY PACS 4.0; GE Healthcare). Both readers were blinded to the presence or nature of an acute ICH. A board-certified neuroradiology consultant with more than 15 years of experience and full access to all clinical and radiological data also classified each scan to provide the ground truth. Acute ICHs were classified as either parenchymal, intraventricular, subarachnoidal (SAH), epidural (EDH), subdural, or a combination, respectively. Each intraparenchymal hematoma (IPH) was also segmented semi-automatically by a neuroradiology resident and validated by the consultant neuroradiologist, based on density thresholds using Amira software (version 5.4.1; Thermo Fisher Scientific Inc.) to provide a reference for the volumes calculated automatically by the algorithm from Brainomix. All segmentations were performed after completing the testing experiment regarding the detection and classification of any ICH. A comparison between the automatically and semi-automatically segmented IPHs is demonstrated in Fig. 1.

### Statistical analysis

Prior to the conduction of the study and creation of TDS, a sample size calculation was performed, based on estimating the desired confidence interval width for the Kappa statistic regarding the agreement between software and ground truth. Assuming Cohen’s kappa coefficient (*κ*) of 0.9 and an expected 50% prevalence of ICH, at least 139 subjects in order for the confidence interval width to be 0.2 were required (i.e., the lower bound of the confidence interval will be 0.8). We decided to include 160 cases (approximately 15% more than the calculated sample size). To enrich the experiment dataset with pathological cases, we intentionally determined a higher ICH prevalence than epidemiological observations.

Specificity and sensitivity were calculated, and a receiver-operating-curve (ROC) analysis was performed for both readers and the algorithm (for ICH and IPH). The interrater agreement against the reference standard was assessed by Cohen’s κ. The intraclass correlation coefficient (ICC) was calculated, using an absolute-agreement definition in a two-way mixed model and a 95% confidence interval, to describe the agreement of quantitative values of IPH between the software and the reference standard. In order to evaluate the similarity between the segmented IPH of both datasets, the Dice coefficient (DC) was calculated.

A standard software package (SPSS 26, IBM) was used for statistical analysis.

## Results

**Fig. 2:**
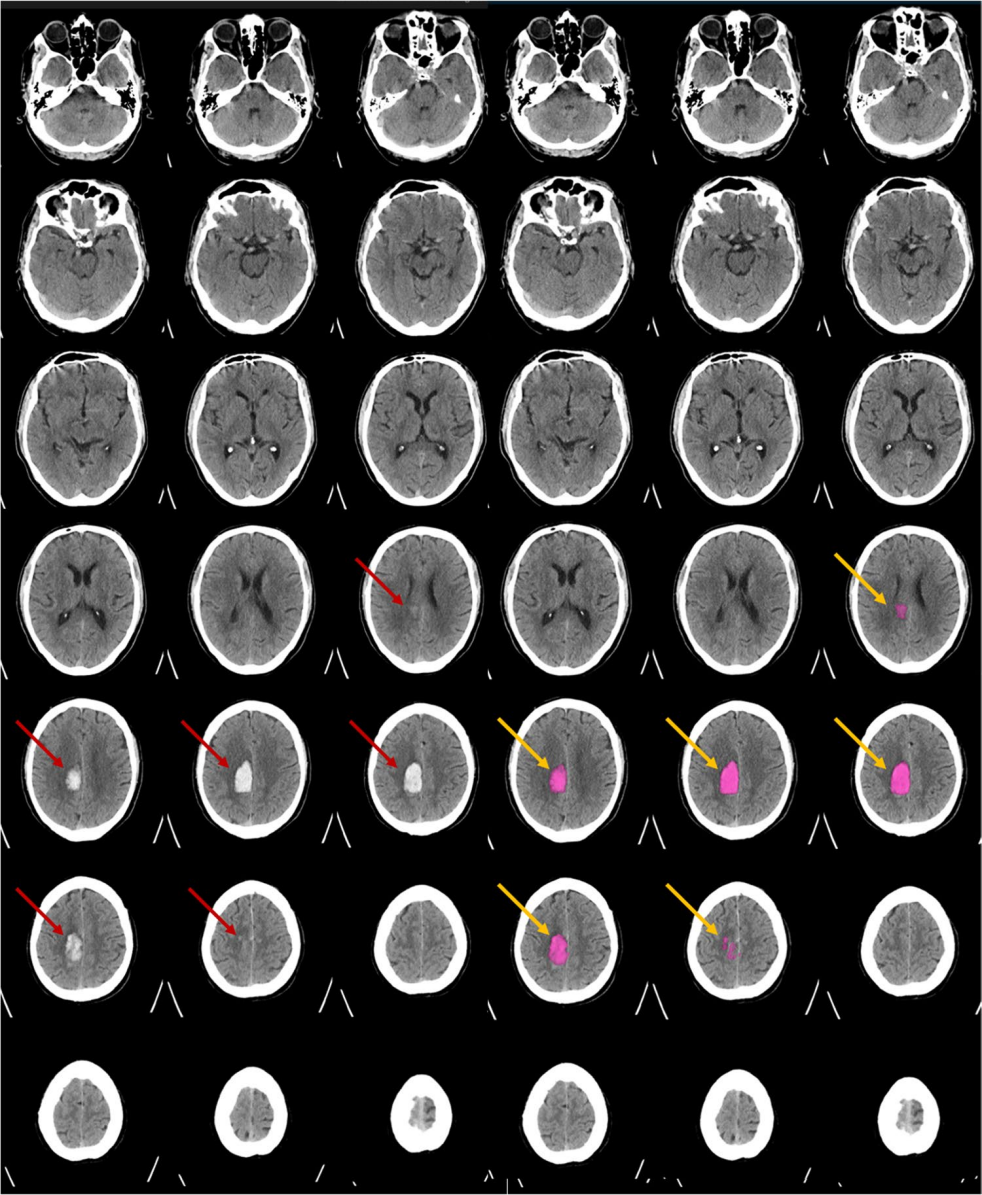
Example of a patient with acute stroke symptoms and intraparenchymal hemorrhage (IPH) on non-contrast-enhanced head CT (left, red arrows). The detection of IPH, segmentation, and volume quantification were performed automatically by the algorithm from Brainomix (right, yellow arrows) in nearly real-time. The above-demonstrated findings will be provided to the user. The fully automated quantification of the IPH in this example resulted in 11 ml

According to the established ground truth, acute ICH was present in 79 of 160 patients, whereby a detailed description of the individual bleeding locations is provided in Table 2 of the Supplementary Appendix. An example for the detection and quantification of an IPH by the algorithm is demonstrated in Fig. 2.

**Fig. 3:**
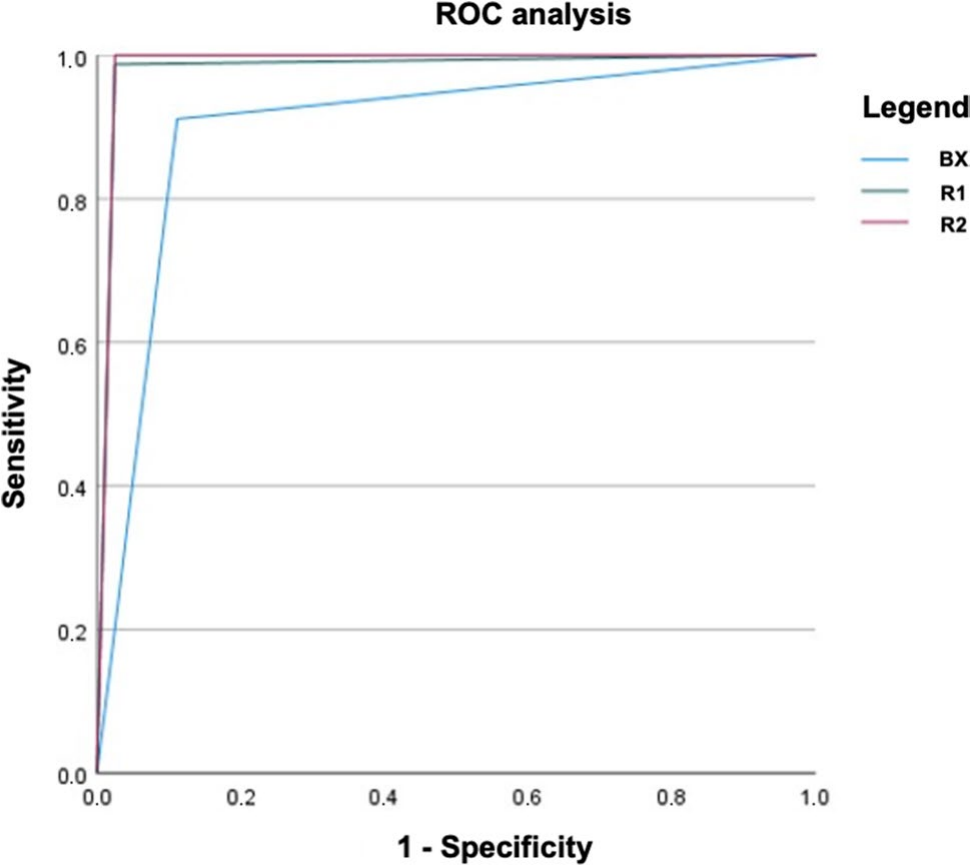
Results of the ROC analysis for detection of intracranial hemorrhage by both readers and the Brainomix algorithm. Further information regarding the sensitivity, specificity, AUC, and Cohen’s kappa coefficient are provided in Table 1. R1 neuroradiology resident 1; R2 neuroradiology resident 2; BX Brainomix algorithm

The results regarding the sensitivity and specificity for detecting any ICH were as follows: Brainomix algorithm: 0.91 and 0.89; reader 1: 0.99 and 0.98; and reader 2: 1.00 and 0.98. The area under the curve (AUC) was 0.90, 0.98, and 0.99, respectively. A detailed description of the results for ICH detection, including the corresponding confidence intervals and Cohen’s kappa coefficient, is provided in Table 1. The results of the ROC analysis are demonstrated in Fig. 3.

**Table 1:** Summary of the results for the detection of any intracranial hemorrhage

	Sensitivity	Specificity	AUC	Kappa	ICC
BX	0.91 (0.83–0.96)	0.89 (0.80–0.95)	0.90 (0.85–0.95)	0.80 (0.71–0.89)	0.80 (0.71–0.89)
R1	0.99 (0.93–1.00)	0.98 (0.91–1.00)	0.98 (0.96–1.00)	0.96 (0.92–1.00)	0.96 (0.92–1.00)
R2	1.00 (0.95–1.00)	0.98 (0.91–1.00)	0.99 (0.97–1.00)	0.98 (0.94–1.00)	0.98 (0.94–1.00)

*BX* Brainomix algorithm; *R1* neuroradiology resident 1; *R2* neuroradiology resident 2; *AUC* area under the curve; *ICC* intraclass correlation coefficient

Confidence intervals are provided in brackets

**Fig. 4:**
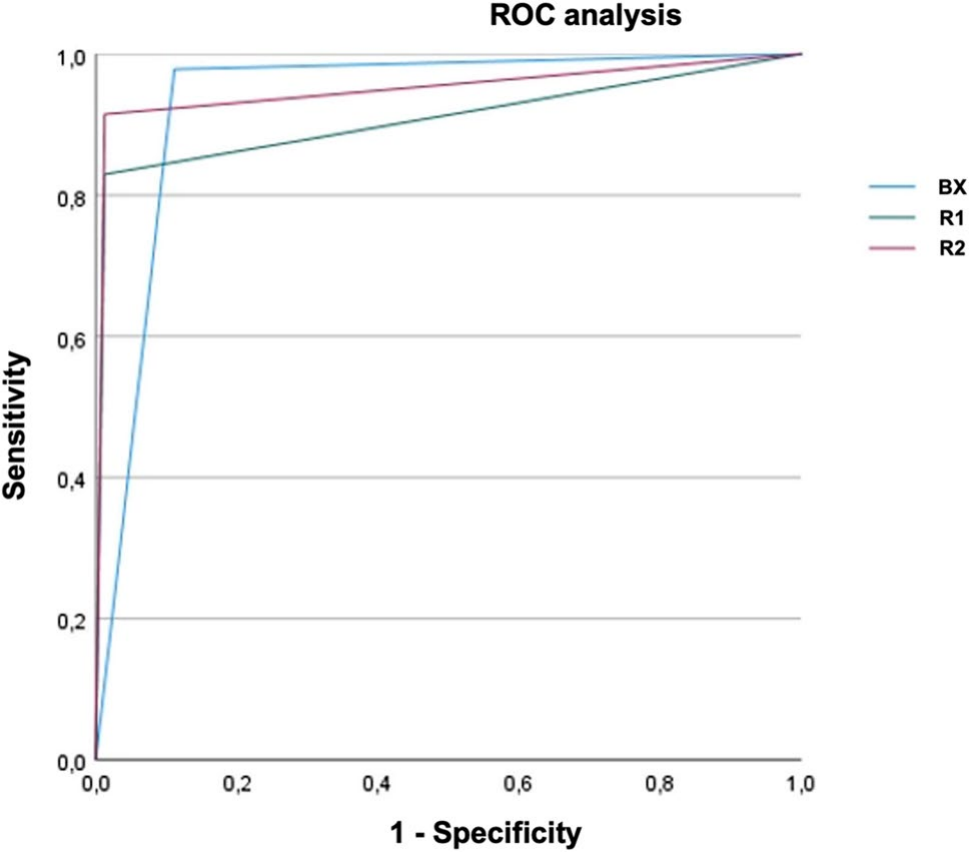
Results of the ROC analysis for detection of intraparenchymal hemorrhage by both readers and the Brainomix algorithm. Further information regarding the sensitivity, specificity, AUC, and Cohen’s kappa coefficient are provided in Table 2. R1 neuroradiology resident 1; R2 neuroradiology resident 2; BX Brainomix algorithm

Sensitivity and specificity for detection of IPH (47 out of 128 cases had IPH; further information is given in Table 4 of the Supplementary Appendix) were as follows: Brainomix algorithm 0.98 and 0.89; reader 1: 0.83 and 0.99; and reader 2: 0.91 and 0.99. AUC was 0.93, 0.91, and 0.95, respectively. A detailed description of the results for IPH detection is demonstrated in Table 2, while the results of the corresponding ROC analysis are shown in Fig. 4.

**Table 2:** Summary of the results for the detection of any intraparenchymal hemorrhage

	Sensitivity	Specificity	AUC	Kappa	ICC
BX	0.98 (0.89–1.00)	0.89 (0.80–0.95)	0.93 (0.89–0.98)	0.84 (0.74–0.93)	0.84 (0.74–0.93)
R1	0.83 (0.69–0.92)	0.99 (0.93–1.00)	0.91 (0.84–0.98)	0.84 (0.75–0.94)	0.84 (0.75–0.94)
R2	0.91 (0.80–0.98)	0.99 (0.93–1.00)	0.95 (0.90–1.00)	0.92 (0.84–0.99)	0.92 (0.84–0.99)

*BX* Brainomix algorithm; *R1* neuroradiology resident 1; *R2* neuroradiology resident 2; *AUC* area under the curve; *ICC* intraclass correlation coefficient

Confidence intervals are provided in brackets

**Fig. 5:**
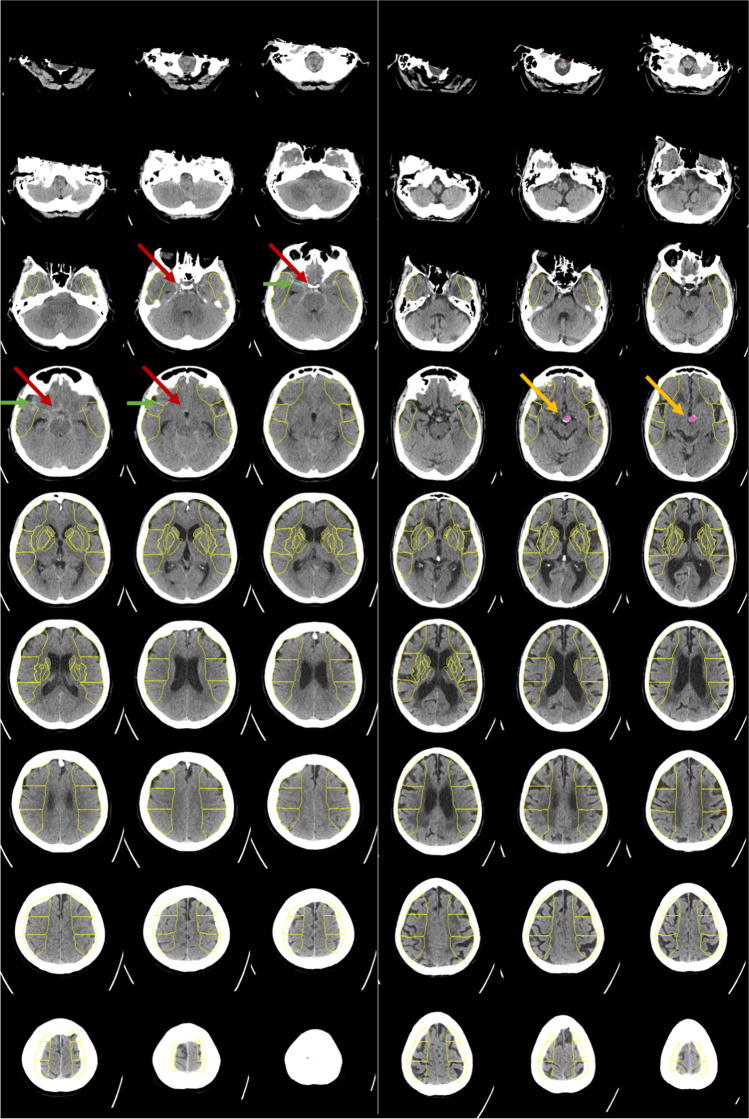
Two examples for patients with acute stroke symptoms for whom the detection of bleeding suspect hyperdense volumes was rated false negative (left) and false positive (right) by the Brainomix algorithm. In the false-negative case, an acute subarachnoid hemorrhage in the basal cisterns (red arrows) was not detected reliably by the algorithm; instead, a hyperdense vessel was marked (green arrows). In the false-positive case, an aneurysm of the basilar artery was detected wrongly as an intracranial bleeding suspect hyperdensity (yellow arrows)

Interreader reliability (IRR) for detection of ICH and IPH showed strong agreements for the algorithm (0.80 and 0.84), reader 1 (0.96 and 0.84), and reader 2 (0.98 and 0.92), respectively. Further information on the ICC results, including the corresponding confidence intervals, is provided in Table 1 (for ICH) and Table 2 (for IPH). There was only one case with EDH in our TDS, and it was correctly detected as an ICH by the algorithm and both neuroradiology residents. Among the 7 false-negative cases of the algorithm were 3 SAH, 3 SDH (2 acute on chronic and 1 acute), and 1 IPH, while 6 of the 9 false-positive cases had calcifications (i.e., 2 calcified meningiomas), and 3 dense vessel signs. Figure 5 depicts two examples of false-positive and false-negative cases.

The ICC of the quantitative IPH volumes of 44 cases was 0.98 (confidence interval: 0.96–0.99) and thus indicating excellent reliability between the algorithm and the semi-automated reference. In 3 IPH cases, a semi-automated segmentation of the hemorrhage volumes was technically not feasible due to its diffuse expansion with additional subarachnoid and intraventricular parts as well as its close proximity to the skull base. Detailed information on the automatically and semi-automatically calculated volumes of each case is given in Table 6 of the Supplementary Appendix.

The mean DC regarding the similarity of the automatic and semi-automatic segmented IPH was 0.82 (0.76–0.87).

## Discussion

For the first time, we describe the performance of the novel AI-based algorithm from Brainomix for the detection and quantification of acute ICH on NCCT of patients with a suspected acute stroke. The algorithm showed a strong agreement for automatic detection of ICH and IPH, respectively, compared to the ground truth. However, the performance of the two neuroradiology residents was better, except for IPH detection, for which the algorithm showed higher sensitivity, but lower specificity. The agreement of the volume measurement of the algorithm and the semi-automated reference was excellent. An overview of several segmentation methods with a comparison to the present study is shown in Table 5 of the Supplementary Appendix.

AI-based image analysis is increasingly applied in clinical practice, especially in the field of acute stroke [13]. Despite this evolution, there are still a limited number of AI-based applications commercially available to assess ICHs in patients with acute stroke. Besides this novel application, two of the clinically most commonly used applications are Rapid ICH by iSchemaView, Inc. and Viz ICH by Viz.ai, Inc. While the results for the algorithm from Brainomix are named above, recent studies for detection of all types of ICHs, excluding hemorrhagic transformations, have demonstrated a sensitivity of 0.95 and a specificity of 0.94 for Rapid ICH (detailed information is provided on https://www.rapidai.com/rapid-ich); studies for Viz ICH have shown a sensitivity and specificity of 0.94 and 0.88 in [14] and 0.90 and 0.99 in [15], respectively (see Table 4 of the Supplementary Appendix).

Moreover, numerous studies regarding the performance of further algorithms for ICH detection [16–22], ICH subtypes classification [20, 22, 23], and segmentation [24–27] have been published in the literature in the last few years. Hssayeni et al. [27] summarized in their research paper various approaches for ICH detection, classification, and segmentation. They noticed that in most studies using large datasets, a high level of sensitivity and specificity could be reached during the testing of the individual algorithms[19–22]. For example, Ye et al. [20] achieved one of the best results. They used a dataset of 2537 CT scans (1642 with ICH and 895 without ICH) for training and tested a dataset of 399 CT scans (194 with ICH and 105 without ICH), resulting in a sensitivity of 0.98 and a specificity of 0.99. A further study by Jnawali et al. [18] included 34,848 CT scans (8465 with ICH and 26,383 without ICH) for training and tested a total of 5509 CT scans (1891 with ICH and 3618 without ICH), obtaining a sensitivity of 0.77 and a specificity of 0.80. Comparing these as well as further findings, investigating commercial and non-commercial software for ICH detection to the Brainomix algorithm, the results feature an equivalent high level of performance [14, 15, 27]. However, a fair comparison between the performances of the individual tools is limited due to the heterogenicity of the training and testing modalities as well as the diversity of the included hemorrhage cases. Moreover, the algorithm’s detection performances varied depending on the type of ICH, while SAH and EDH were the most difficult types to classify [20–26]. The described difficulty in detecting SAH is in line with our results; however, EDH cases were only occasionally presented in our neurological emergency department, as trauma cases were primarily treated by the neurosurgical emergency department. Nevertheless, acute SDHs were also challenging to recognize in our study, matching the literature and the common difficulties in SDH diagnosis [28]. Regarding the studies investigating the performance of the commercially available applications Rapid ICH and VIZ ICH, one major strength of our dataset is the inclusion of various types of ICHs and other intracranial hyperdensities, while the other studies provided no further information to this regard [14, 15] (additional reference: https://www.rapidai.com/rapid-ich).

In Table 4 of the Supplementary Appendix, we recapitulated some results based on Hssayeni et al.’s research and compared their findings to the present study as well as to both other commercially available ICH detection tools.

Some limitations of our study should be noted and discussed. In concordance with our study design, cases with significant artifacts were excluded; this may not reflect all possible real-work circumstances. The usage of a single CT scanner for the imaging process may have reduced the technical bias; however, a generalization of the results to other types of CT scanners may be limited. Blinding essential clinical data—like the side of hemisymptomatic neurological deficiency—might have influenced the performance of the physicians; the clinical presentation of symptoms is usually essential for the anatomical localization of pathologies on radiological images.

Moreover, our study focused mainly on non-traumatic ICHs, and all the collected images were obtained from patients with a suspected acute ischemic or hemorrhagic stroke. Therefore, the algorithm’s performance in detecting and quantifying ICHs in traumatic cases necessitates further investigation.

Despite these limitations, the present study delivered concrete results of the algorithm's sensitivity and specificity compared to neuroradiology residents and the ground truth. Even with the presence of challenging, multiple non-pathological hemorrhage-like structures in our TDS, the algorithm’s performance was comparable to other commercial and non-commercial algorithms used for ICH and IPH detection, while resulting in excellent findings regarding the quantification of IPH. Despite the good results achieved by the Brainomix algorithm, the overall performance of neuroradiologists was better. Therefore, the algorithm’s current performance precludes its use as a stand-alone automated tool for establishing a final diagnosis and selecting stroke patients for reperfusion treatment; however, the aim of AI-powered software tools to interpret images of stroke victims should rather be to enhance the performance of physicians and more studies to this regard are needed [29]. Moreover, the excellent automated volume quantification might enable a standardized prospective patient selection in future IPH trials.

## Conclusion

In this dataset with 160 cases enriched with challenging non-ICH pathologies, the AI-based algorithm from Brainomix was reliable in detecting acute ICHs and quantifying IPH-volumes. This actual performance may help to provide a well-founded second opinion for clinicians before establishing a final diagnosis but precludes the algorithm to be used as a stand-alone tool. Moreover, larger, randomized controlled trials with a prospective study design should be performed to validate the findings presented here.

## Supplementary Information

Below is the link to the electronic supplementary material.

Supplementary file1 (DOCX 348 KB)

## Abbreviations

AI: Artificial intelligence
ASPECTS: Alberta Stroke Program Early Computed Tomography Score
AUC: Area under the curve
CAD: Computer-aided diagnosis
DC: Dice coefficient
DNN: Deep neural networks
EDH: Epidural hematoma
ICC: Intraclass correlation coefficient
ICH: Intracranial hemorrhage
IPH: Intraparenchymal hematoma/hemorrhage
IRR: Interreader reliability
NCCT: Non-contrast-enhanced head computed tomography
NIHSS: National Institutes of Health Stroke Scale
pmRS: Pre-stroke Rankin Score
ROC: Receiver-operating-curve
SD: Standard deviation
TDS: Testing data-subset

## Declarations

### Guarantor

The scientific guarantor of this publication is Simon Nagel.

### Statistics and biometry

One of the authors (SG) has significant statistical expertise.

### Methodology

• Retrospective

• Diagnostic study

• Performed at one institution
