# Supplementary material for: Automated detection and segmentation of intracranial hemorrhage suspect hyperdensities in non-contrast-enhanced CT scans of acute stroke patients
Source: Eur Radiol. 2021 Nov 13;32(4):2246–54. doi: 10.1007/s00330-021-08352-4 (PMC8921016; doi:10.1007/s00330-021-08352-4)
Supplement: Supplementary file 1 — Supplementary file1 (DOCX 348 KB) [file 330_2021_8352_MOESM1_ESM.docx]

**Supplementary Appendix**

**Index**

Supplementary Tables

Table 1 Strata used during the systematic stratified convenience sampling

Table 2 Detailed description of the individual bleeding locations

Table 3 Breakdown of our test results

Table 4 Overview of different methods for ICH detection and comparison of the findings to the present study

Table 5 Overview of different methods of ICH segmentation and comparison of the results to the present study

Table 6 Detailed description of the calculated IPH volumes

Supplementary Figures

Figure 1 Resume of the inclusion criteria and the sampling process

**Supplementary Tables**

**Table 1**

Strata used during the systematic stratified convenience sampling

| **Main groups** | **Subgroups** | **Aimed number of cases** |
| --- | --- | --- |
| 1. Cases without intracranial hemorrhage | 1 a. pronounced physiological or pathological intracranial calcifications | 15 |
|  | 1 b. hemorrhage-like structures (e.g., meningioma, hyperdense vessel sign, aneurysma and tumors) | 15 |
|  | 1 c. pronounced brain tissue defects | 10 |
|  | 1 d. normal findings without 1 a, 1 b and 1 c | 40 |
| 2. Cases with intracranial hemorrhage | 2 a. subarachnoidal hemorrhage | 20 |
|  | 2 b. basal ganglia hemorrhage | 15 |
|  | 2 c. lobar hemorrhage | 15 |
|  | 2 d. subdural hematoma and epidural hematoma | 10 |
|  | 2 e. infratentorial, including mixed infra- and supratentorial | 10 |
|  | 2 f. mixed hemorrhage, excluding 2 e | 10 |

**Table 2**

Detailed description of the individual bleeding locations

IPH intraparenchymal hemorrhage

SAH subarachnoidal hemorrhage

SDH subdural hemorrhage

IVH intraventricular hemorrhage

EDH epidural hematoma

Infra infratentorial

Supra supratentorial

| Cases | IPH1 / SAH2 / SDH3 /  IVH4 / EDH5 | Infra 1  Supra 2 | Right 1  Left 2 | Localization |
| --- | --- | --- | --- | --- |
| ICH003 | 2 | 2 | 1; 2 | suprasellar, perisylvic |
| ICH004 | 1 | 2 | 2 | Frontal, basal ganglia |
| ICH005 | 1 | 2 | 1 | frontal |
| ICH009 | 1 | 2 | 2 | frontal |
| ICH010 | 2 | 2 | 1 | sulcus centralis |
| ICH011 | 2 | 2 | 1; 2 | suprasellar, perisylvic |
| ICH012 | 3 | 2 | 1 | temporal |
| ICH013 | 1 | 2 | 2 | temporal |
| ICH014 | 2 | 1; 2 | 1; 2 | perimesencephalic, suprasellar |
| ICH015 | 2 | 2 | 0 | suprasellar |
| ICH017 | 1 | 2 | 2 | basal ganglia |
| ICH022 | 3 | 2 | 1 | fronto-temporal |
| ICH024 | 2 | 2 | 1; 2 | suprasellar, perisylvic |
| ICH026 | 4 | 2 | 0 | third ventricle |
| ICH028 | 2; 1 | 2 | 1 | temporo-parietal |
| ICH030 | 4 | 2 | 1 | occipital |
| ICH032 | 3 | 2 | 0 | frontal, both sides |
| ICH035 | 2; 1 | 2 | 2 | massive hemispheric hemorrhage |
| ICH037 | 1 | 2 | 2 | Temporal, parietal |
| ICH040 | 5 | 2 | 2 | frontal |
| ICH041 | 1 | 2 | 2 | frontal, parietal |
| ICH042 | 1 | 1 | 0 | pons |
| ICH043 | 1; 2 | 1 | 2 | cerebellum |
| ICH046 | 2 | 1; 2 | 1; 2 | perimesencephalic, suprasellar |
| ICH047 | 2 | 2 | 0 | frontoparietal |
| ICH048 | 3 | 2 | 1 | frontal |
| ICH055 | 2 (1) | 2 | 0 | suprasellar, perisylvic |
| ICH057 | 1; 4 | 2 | 1 | basal ganglia, ventricular |
| ICH058 | 1; 4 | 2 | 1 | thalamic, ventricular |
| ICH059 | 3 | 2 | 1; 2 | bihemispheric |
| ICH061 | 1; 2; 4 | 1 | 0 | nucleus dentatus R, ventricular |
| ICH063 | 1 | 2 | 1 | parietal |
| ICH064 | 2 | 2 | 1; 2 | suprasellar, perisylvic |
| ICH065 | 1 | 2 | 2 | occipital |
| ICH068 | 2 | 2 | 1; 2 | suprasellar, perisylvic |
| ICH072 | 1 | 2 | 0 | Parietal both sides |
| ICH076 | 1; 4 | 2 | 1 | basal ganglia, ventricular |
| ICH078 | 1 | 2 | 1 | frontal |
| ICH082 | 3 | 2 | 2 | hemispherical |
| ICH084 | 1 | 1 | 0 | pons |
| ICH085 | 1 | 1 | 0 | pons |
| ICH087 | 1 | 2 | 1 | frontal |
| ICH091 | 1; 4 | 2 | 2 | basal ganglia, ventricular |
| ICH094 | 2 | 2 | 1; 2 | insular dorsal |
| ICH095 | 3 | 2 | 2 | frontal |
| ICH097 | 1; 4 | 2 | 2 | basal ganglia, ventricular |
| ICH098 | 1; 3 | 2 | 1 | temporal |
| ICH099 | 1 | 2 | 2 | basal ganglia |
| ICH100 | 2 | 2 | 1; 2 | suprasellar, perisylvic |
| ICH101 | 1 | 2 | 1 | basal ganglia |
| ICH103 | 2 | 1; 2 | 1; 2 | perimesencephalic, suprasellar |
| ICH105 | 1; 4 | 2 | 1 | basal ganglia, ventricular |
| ICH106 | 2 | 2 | 1; 2 | suprasellar, perisylvic |
| ICH110 | 1 | 2 | 1 | putamen |
| ICH112 | 1 | 2 | 1 | thalamic |
| ICH113 | 2 | 2 | 1; 2 | suprasellar, perisylvic |
| ICH114 | 1; 4 | 2 | 1 | frontal, ventricular |
| ICH115 | 1 | 2 | 2 | basal ganglia |
| ICH116 | 1 | 2 | 1 | temporal |
| ICH117 | 1; 4 | 2 | 2 | frontal, ventricular |
| ICH118 | 1; 4 | 1 | 2 | cerebellar, ventricular |
| ICH120 | 2 | 2 | 0 | massive SAH |
| ICH124 | 1 | 1 | 0 | vermis |
| ICH125 | 2 | 2 | 2 | sulcus praecentralis |
| ICH126 | 1; 4 | 1 | 1 | basal ganglia |
| ICH127 | 2 | 2 | 1; 2 | suprasellar, perisylvic |
| ICH128 | 2 | 2 | 2 | sulcus frontalis |
| ICH130 | 1 | 1 | 2 | cerebellum |
| ICH131 | 1; 2 | 1 | 2 | temporal |
| ICH132 | 1 | 1 | 0 | vermis, Pons |
| ICH134 | 2 | 2 | 1; 2 | suprasellar, perisylvic |
| ICH136 | 1 | 2 | 2 | basal ganglia (very small) |
| ICH140 | 1 | 2 | 2 | basal ganglia |
| ICH143 | 1; 4 | 2 | 2 | basal ganglia, ventricular |
| ICH147 | 3 | 2 | 0 | both sides |
| ICH148 | 4; 1 | 2 | 2 | occipital |
| ICH150 | 3 | 2 | 2 | frontal |
| ICH154 | 1 | 2; 1 | 1; 2 | paracentral, nucleus dentatus |
| ICH159 | 1 | 2 | 1 | temporal |

**Table 3**

Breakdown of our test results

|  | TP | TN | FP | FN |
| --- | --- | --- | --- | --- |
| ICH-BX | 72 | 72 | 9 | 7 |
| ICH-R1 | 78 | 79 | 2 | 1 |
| ICH-R2 | 79 | 79 | 2 | 0 |
| IPH-BX | 46 | 72 | 9 | 1 |
| IPH-R1 | 39 | 80 | 1 | 8 |
| IPH-R2 | 43 | 80 | 1 | 4 |

BX Brainomix algorithm

R1 Neuroradiology resident 1

R2 Neuroradiology resident 2

TP True positive

TN True negative

FP False positive

FN False negative

**Table 4**

An overview based on the review of Hssayeni et al.[1] regarding the different methods for ICH detection proposed in various studies and a comparison of their findings to ours as well as to other commercially available tools.

^a^ CNN: Convolutional Neural Networks

^b^ RF: Random Forest

^c^ RNN: Recurrent neural network

^d^ ROI: Regions of interest

^#^ Information provided by the company on its official website

| **References**  **(Included acute hemorrhage types)** | **Number of CT scans**  **used for testing** | |  | **ICH detection results** | | |
| --- | --- | --- | --- | --- | --- | --- |
|  | with ICH (all types) | without ICH | Method | Sensitivity (%) | Specificity  Precision*  (%) | AUC |
| Our study | 79 | 81 | CNN^a^ (Brainomix Ltd.) | 91 | 89 | 0.90 |
|  |  |  | Reader 1 | 99 | 98 | 0.98 |
|  |  |  | Reader 2 | 100 | 98 | 0.99 |
| RapidAI ^#^ | n/a | n/a | 2D/3D CNN  (Rapid ICH; iSchemaView, Inc.) | 95 | 94 | n/a |
| Yuh et al. [2] | 52 | 158 | Threshold-based | 98 | 59 | n/a |
| Prevedello et al. [3] | 50 | 35 | CNN | 90 | 85 | 0.91 |
| Jnawali et al. [4] | 1891 | 3618 | CNN | 77 | 80* | 0.87 |
| Chilamkurthy et al. [5] | 2494 + 205 | 18601 + 286 | CNN (ResNet18) and RF^b^ | 92 | 70 | 0.93 |
| Ye et al.[6] | 194 | 105 | 3D joint CNN-RNN^c^ | 98 | 99 | 1.00 |
| Chang et al. [7] | 82 | 780 | ROI^d^-based CNN | 95 | 97 | 0.97 |
| Lee et al. [8] | 100 + 107 | 100 + 130 | CNN | 95 | 95 | 0.98 |
| **References** | **Number of CT scans**  **used for testing** | |  | **IPH Detection Results** | | |
|  | with IPH | without IPH | Method | Sensitivity (%) | Specificity  (%) | AUC |
| Our Study | 47 | 81 | CNN (Brainomix Ltd.) | 98 | 89 | 0.93 |
|  |  |  | Reader 1 | 83 | 99 | 0.91 |
|  |  |  | Reader 2 | 91 | 99 | 0.95 |
| Mohammaden et al. [9] | 163 | 48 | CNN (Viz ICH v1.4; Viz.ai, Inc.) | 94 | 88 | 0.94 |
| Barreira et al. [10] | 132 | 152 | CNN (Viz ICH v2.0; Viz.ai, Inc.) | 90 | 99 | 0.96 |

**Table 5**

An overview based on the review of Hssayeni et al. [1] regarding different methods of ICH segmentation proposed in various studies and a comparison of their results to the present study.

^a^ CNN: Convolutional Neural Networks

^b^ ROI: Regions of interest

| **References** | **Number of segmented ICHs/IPHs** | **Ground truth** | **Evaluated ICH segmentation method** | **Dice coefficient** |
| --- | --- | --- | --- | --- |
| Our study | 44 | Semi-automatic segmentation using Amira software (Thermo Fisher Scientific Inc.) | CNN^a^ (Brainomix Ltd.) | 0.82 (IPH) |
| Bhadauria et al. [11] | 20 | manually delineated hemorrhage region drawn by an expert | Fuzzy c-mean clustering  region-based active contour | 0.87 |
| Muschelli et al. [12] | 102 | Manual segmentation using OsiriX imaging software (OsiriX v. 4.1, Pixmeo) | Logistic regression  Logistic regression with LASSO  Generalized additive model  Random forest classifier | 0.89 |
| Kuang et al. [13] | 30 | Manual segmentation using the ITK-SNAP software | semi-automated segmentation approach (Semi-D-Unet) based on U-Net (deep Fully Convolutional Networks) | 0.65 |
| Chang et al. [7] | 82 | Manual segmentation | ROI^b^-based CNN | 0.85 (Average)  0.93 (IPH)  0.86 (EDH/SDH)  0.77 (SAH) |

**Table 6**

Detailed description of the automatically (by the Brainomix algorithm) and the semi-automatically (ground truth) calculated volumes of all 44 cases with intraparenchymal hemorrhage (IPH). Volumes are demonstrated in ml.

| Cases | IPH-volumes calculated automated by the Brainomix algorithm | IPH-volumes calculated semi-automated (ground truth) |
| --- | --- | --- |
| ICH004 | 147.533 | 123.59 |
| ICH005 | 11.796 | 9.81 |
| ICH009 | 157.104 | 135.31 |
| ICH013 | 1.702 | 1.52 |
| ICH017 | 8.27 | 6.30 |
| ICH028 | 114.546 | 77.67 |
| ICH037 | 67.539 | 58.49 |
| ICH041 | 71.793 | 50.80 |
| ICH042 | 4.464 | 6.96 |
| ICH043 | 27.809 | 45.67 |
| ICH057 | 86.839 | 80.21 |
| ICH058 | 43.622 | 59.67 |
| ICH061 | 28.354 | 31.37 |
| ICH063 | 35.74 | 30.36 |
| ICH065 | 3.299 | 8.44 |
| ICH072 | 2.164 | 4.32 |
| ICH076 | 37.461 | 34.18 |
| ICH078 | 60.864 | 34.29 |
| ICH084 | 1.705 | 2.97 |
| ICH085 | 4.435 | 5.74 |
| ICH087 | 9.748 | 7.19 |
| ICH091 | 28.069 | 21.01 |
| ICH097 | 39.088 | 34.26 |
| ICH098 | 104.302 | 95.15 |
| ICH099 | 1.489 | 3.58 |
| ICH101 | 14.219 | 13.38 |
| ICH105 | 165.348 | 153.74 |
| ICH110 | 17.144 | 13.75 |
| ICH112 | 13.837 | 13.76 |
| ICH114 | 31.409 | 55.17 |
| ICH115 | 27.991 | 25.13 |
| ICH116 | 0 | 0.14 |
| ICH117 | 120.499 | 101.90 |
| ICH118 | 76.129 | 49.71 |
| ICH124 | 52.443 | 48.49 |
| ICH126 | 75.336 | 68.17 |
| ICH130 | 4.094 | 11.84 |
| ICH132 | 10.68 | 8.88 |
| ICH136 | 0.249 | 0.80 |
| ICH140 | 17.453 | 18.07 |
| ICH143 | 46.316 | 42.63 |
| ICH148 | 76.228 | 67.41 |
| ICH154 | 20.463 | 18.38 |
| ICH159 | 44.644 | 39.91 |

**Supplementary Figures**

**Figure 1**

**
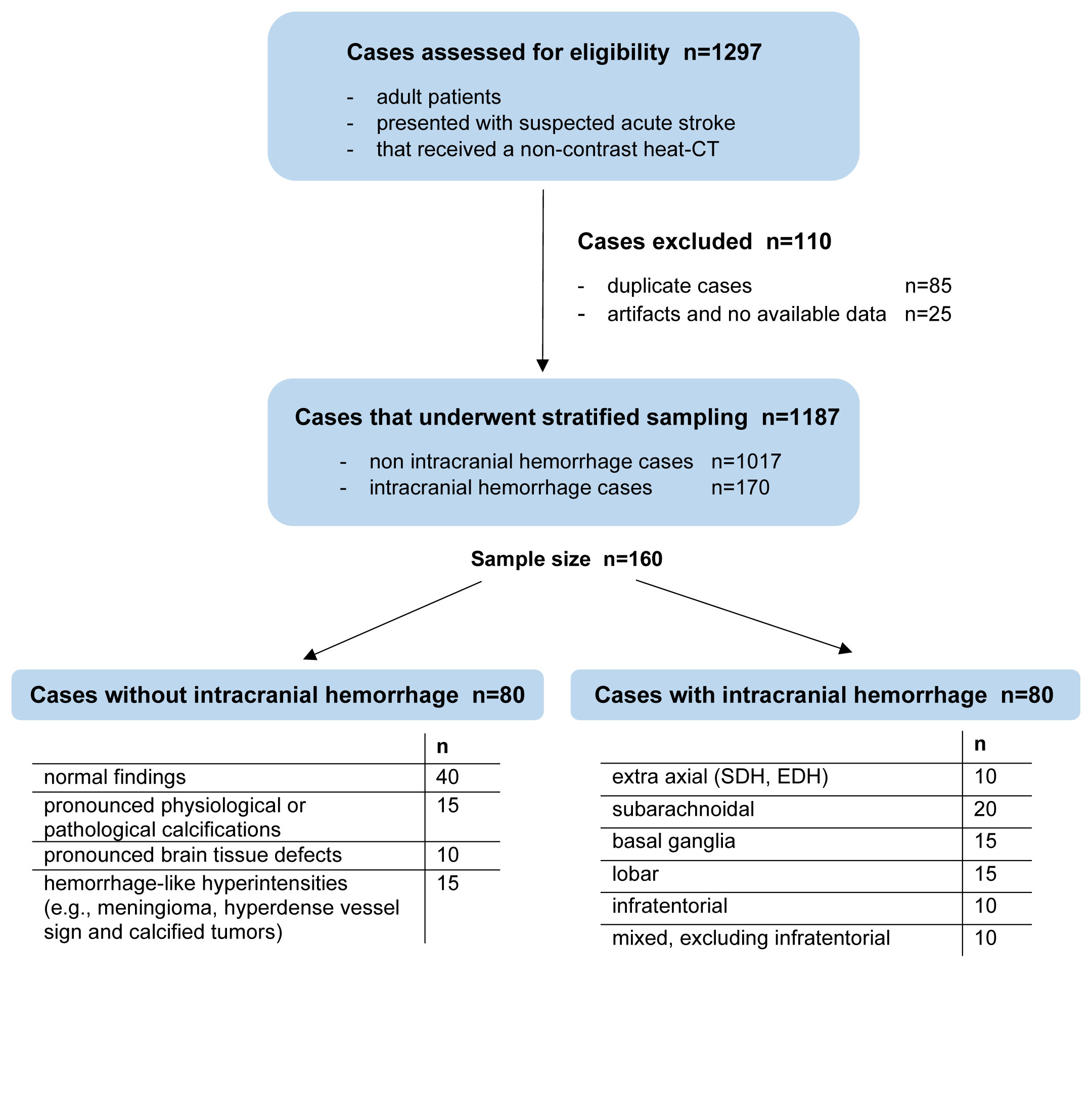
**Flow chart summarizing the inclusion criteria and sampling process.

**References**

1 Hssayeni MD, Croock MS, Salman AD, Al-khafaji HF, Yahya ZA, Ghoraani B (2020) Intracranial Hemorrhage Segmentation Using a Deep Convolutional Model. Data 5:14

2 Yuh EL, Gean AD, Manley GT, Callen AL, Wintermark M (2008) Computer-Aided Assessment of Head Computed Tomography (CT) Studies in Patients with Suspected Traumatic Brain Injury. Journal of Neurotrauma 25:1163-1172

3 Prevedello LM, Erdal BS, Ryu JL et al (2017) Automated Critical Test Findings Identification and Online Notification System Using Artificial Intelligence in Imaging. Radiology 285:923-931

4 Jnawali K, Mohammad RA, Navalgund R, Alpen APMD (2018) Deep 3D convolution neural network for CT brain hemorrhage classificationProcSPIE,

5 Chilamkurthy S, Ghosh R, Tanamala S et al (2018) Deep learning algorithms for detection of critical findings in head CT scans: a retrospective study. The Lancet 392:2388-2396

6 Ye H, Gao F, Yin Y et al (2019) Precise diagnosis of intracranial hemorrhage and subtypes using a three-dimensional joint convolutional and recurrent neural network. Eur Radiol 29:6191-6201

7 Chang PD, Kuoy E, Grinband J et al (2018) Hybrid 3D/2D Convolutional Neural Network for Hemorrhage Evaluation on Head CT. American Journal of Neuroradiology 39:1609

8 Lee H, Yune S, Mansouri M et al (2019) An explainable deep-learning algorithm for the detection of acute intracranial haemorrhage from small datasets. Nature Biomedical Engineering 3:173-182

9 Mohammaden M, Pisani L, Camara CPd et al (2020) Abstract TP90: Advance: Automated Detection and Volumetric Assessment of Intracerebral Hemorrhage. Stroke 51:ATP90-ATP90

10 Barreira CM, Rahman HA, Bouslama M et al (2018) Advance study: automated detection and volumetric assessment of intracerebral hemorrhage. European Stroke Journal European Stroke Organisation Conference: Abstracts 3:3–204

11 Bhadauria HS, Dewal ML (2014) Intracranial hemorrhage detection using spatial fuzzy c-mean and region-based active contour on brain CT imaging. Signal, Image and Video Processing 8:357-364

12 Muschelli J, Sweeney EM, Ullman NL, Vespa P, Hanley DF, Crainiceanu CM (2017) PItcHPERFeCT: Primary Intracranial Hemorrhage Probability Estimation using Random Forests on CT. NeuroImage: Clinical 14:379-390

13 Kuang H, Menon BK, Qiu W (2019) Segmenting Hemorrhagic and Ischemic Infarct Simultaneously From Follow-Up Non-Contrast CT Images in Patients With Acute Ischemic Stroke. IEEE Access 7:39842-39851
